# MRI findings in a case of spinal cord Wallerian degeneration following trauma

**DOI:** 10.17712/nsj.2016.4.20160278

**Published:** 2016-10

**Authors:** Puneet Mittal, Ranjana Gupta, Amit Mittal, Kapish Mittal

**Affiliations:** *Department of Radiodiagnosis, Maharishi Markandeshwer Institute of Medical Sciences and Research, Mullana, Haryana, India*

Wallerian degeneration is a well-known entity which represents distal axonal degeneration and myelin disintegration following interruption of any axon from its cellular connection. It was first described by Weller in 1850 and is named after him.^1^ It is well described entity on imaging in brain but is less well described in spinal cord with only few reports in radiological literature and in post mortem pathological studies.^1^-^4^ We described sequential magnetic resonance imaging (MRI) findings in a case of spinal cord injury with development of changes of Wallerian degeneration in delayed scan.

**Figure 1:**
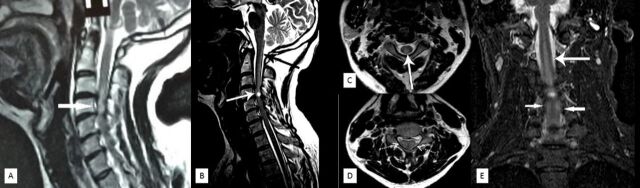
Sagittal T2W **a**) image of intial MRI at the time of injury shows cord contusion at C3 to C4-5 levels appearing hyperintense (White arrow), **b**, **a**) image of MRI done after 6 months with sharpened contrast shows focal syrinx at C4-5 level (white arrow) with long segment abnormal hyperintense signal in dorsal column superiorly extending upto cervicomedullary junction (black arrow). Axial T2W **c**) at C2-3 level showing hyperintense signal in dorsal column (white arrow). **d**) image at C6-7 level showing hyperintense signal in lateral cortico-spinal tracts (dotted white arrows). Coronal STIR images **e**) showing syrinx at C4-5 level with wallerian degeneration changes cranial and caudal to it (white arrows).

A 35-year-old male patient presented for MRI. He had history of fall 6 months back that was followed by quadriparesis with reduced power in bilateral upper and lower limbs (3/5). Magnetic resonance imaging at that time showed cord contusion with hyperintense signal in spinal cord at C3 to C4-5 levels with associated degenerative disc disease. No fracture or dislocation was seen (**Figure 1**). Patient was managed conservatively. He now presented with paresthesias in bilateral lower limbs and reduced grip power in bilateral upper limbs that was worsened for last one month. Magnetic resonance imaging showed small focal syrinx in the cord at C4-5 level. Abnormal signal was seen in the dorsal column of spinal cord extending superiorly with extension upto cervico-medullary junction. Hyperintense signal was also seen in bilateral lateral corticospinal tracts inferior to the level of syrinx extending up to C7 vertebral level. Based on the characteristic findings, a diagnosis of post traumatic Wallerian degeneration of spinal cord was made.

Four stages of Wallerian degeneration in brain were described by Kuhn in brain in 1989.^1^,^4^ In the first stage in first four weeks, there is degeneration of axons without any significant change in the myelin sheath and normal signal intensity on MR imaging. Stage II lasts for 4-14 weeks and is characterized by myelin protein breakdown process with relative preservation of myelin lipids and is characterized by T2W hypointense signal on MRI. Stage III occurs after 14 weeks and is characterized by lipid breakdown in myelin with gliosis and increased water content and shows hyperintense signal on T2W images. Stage IV occurs years after the injury and is characterized by atrophic changes with persistence of hyperintense signal. These stages described for brain changes are expected to be similar in spinal cord Wallerian degeneration. These stages were described prior to 1990 when conventional spin echo (CSE) was used for imaging acquisition of T2W images. After 1990, fast spin echo (FSE) technique became the mainstay for acquisition of T2W images and therefore the above signal intensities may not be completely reproducible.

There is a fundamental difference in Wallerian degeneration in central nerve system (CNS) when compared to peripheral nervous system (PNS). In PNS, the axonal injury is followed by rapid focal invasion of macrophages from systemic circulation and focal proliferation of Schwann cells along the myelin sheaths. These rapidly breakdown and engulf the degenerated axons and myelin sheath and the whole process is complete within a period of approximately three weeks and this creates and environment which is conducive to axonal regeneration. On the other hand, in CNS, there is no invasion of systemic macrophages in the CNS, thereby delaying myelin breakdown. In initial phase after injury, endogenous proteases like caplains which are leaked from the injured axons itself initiate the axonal and peri-axonal myelin degeneration. The myelin cells of CNS, oligodendrocytes, undergo progressive slow apoptotic death which release neurite outgrowth inhibitor, NOGO-A and inhibit neural growth. Therefore, Wallerian degeneration in CNS is followed by gliotic reaction and eventual scarring and volume loss with no attempt at regeneration.^1^,^3^,^4^

In spinal cord injury, Wallerian degeneration is seen in dorsal column cranial to the site of injury and in lateral corticospinal tracts below the level of injury. The length of involved segment is proportional to the number of axons that are damaged. Therefore, the length is more in cervical region, where the axons are more tightly packed as compared to dorsal or lumbar region.^3^,^4^ The changes of Wallerian degeneration can be detected earlier using diffusion tensor imaging (DTI) even when no signal changes are seen on conventional imaging. Reduced fractional anisotropy (FA) values both above and below the level of injury have been demonstrated with DTI that represents Wallerian degeneration. Early detection of Wallerian degeneration by DTI may have important implications in future as stem cell therapy is being used in spinal injury cases and DTI could be potentially useful for detecting early signs of regeneration.

The main radiological differential diagnosis for this condition is sub-acute post-traumatic ascending myelopathy. It is an entity of unknown etiology however venous ischemic, viral infections and changes in cerebro-spinal fluid (CSF) dynamics have been proposed as a potential etiology of this condition. It presents with delayed onset ascending neurological deficit more than 4 spinal levels above the original site of injury. It usually presents within 3 weeks but can present up to 3 months from injury and may be subclinical and detected only on imaging. It presents with cord swelling and central cord T2W hyper intensity in acute phase with atrophic changes in delayed phase images.^5^ These changes are different from Wallerian degeneration in which changes are seen in the dorsal column superior to the site of injury and in the lateral cortico-spinal tracts inferior to the site of injury. Another cause of T2W hyper intensity in the dorsal column is subacute combined degeneration secondary to vitamin B12 deficiency, however clinical presentation and context is different and therefore it does not pose any problems in radiological differential diagnosis.

In conclusion, we present sequential MRI findings of wallerian degeneration in cervical spinal cord where initial injury was followed by worsening of symptoms and new areas of signal abnormality on MR imaging which were consistent with changes of Wallerian degeneration.
